# Carotid Intima-Media Thickness, Overweight, and Poor Sleep Quality in Commercial Airline Pilots: A Cross-Sectional Study

**DOI:** 10.7759/cureus.63552

**Published:** 2024-07-01

**Authors:** Piercarlo Minoretti, Andrés Santiago Sáez, Ángel García Martín, Miryam Liaño Riera, Manuel Gómez Serrano

**Affiliations:** 1 Occupational Health, Studio Minoretti, Oggiono, ITA; 2 Legal Medicine, Hospital Clinico San Carlos, Madrid, ESP; 3 Legal Medicine, Psychiatry, and Pathology, Complutense University of Madrid, Madrid, ESP

**Keywords:** ultrasound, intima-media thickness, overweight, sleep, atherosclerosis, airline pilots

## Abstract

Background

Cardiovascular disease is a leading cause of premature career termination in commercial airline pilots (APs). In this cross-sectional study, we sought to investigate the relationship between intima-media thickness (IMT), a marker of subclinical atherosclerosis, and cardiovascular risk factors in APs, focusing on overweight status and sleep quality.

Methods

A total of 140 male APs were categorized into four groups based on body mass index (BMI) and Pittsburgh Sleep Quality Index (PSQI) score: overweight poor sleepers (OW-PS), overweight good sleepers (OW-GS), normal weight poor sleepers (NW-PS), and normal weight good sleepers (NW-GS). IMT was quantified in the common carotid artery (CCA) and carotid bulb using ultrasound, yielding a composite IMT (IMTcom) measure. Common cardiovascular risk factors were assessed in all participants.

Results

The prevalence of overweight and poor sleep quality was 43.6% and 32.9%, respectively. The OW-PS group had significantly higher age, heart rate, total cholesterol, and low-density lipoprotein (LDL) cholesterol compared to other groups (p<0.05). Overweight pilots, regardless of sleep quality, had increased IMTcom compared to normal-weight pilots (p<0.001). Age and LDL cholesterol were independent predictors of IMTcom in the OW-PS and OW-GS groups (p<0.05).

Conclusions

Overweight status, irrespective of sleep quality, is associated with increased IMT in APs, suggesting a higher burden of subclinical atherosclerosis. Interventions focused on reducing LDL cholesterol levels and managing age-related cardiovascular risk factors could be advantageous in mitigating the risk of atherosclerotic vascular disease in overweight pilots.

## Introduction

As early as 1992, McCall et al. [1] identified cardiovascular disease (CVD) as the primary cause of premature career termination for commercial airline pilots (APs), accounting for a significant proportion of all medical causes combined. Despite the widespread use of cardiovascular risk prediction scores by aviation authorities globally, Wirawan et al. [2] found that these tools have low sensitivity and fail to predict nearly half of cardiovascular events. Furthermore, the same research group highlighted the limitations of exercise electrocardiograms (ECG) as a diagnostic test for detecting cardiovascular risk in APs and suggested that the coronary artery calcium score may enhance risk stratification [3]. Additionally, they emphasized the importance of aggressively managing hyperglycemia, hyperlipidemia, and hypertension in asymptomatic pilots with a five-year CVD risk of 5-10% and 10-15% [4].

Prior studies have consistently shown a high prevalence of cardiometabolic risk factors among APs. For instance, Alaminos-Torres et al. [5] reported that 53.6% of 304 Spanish male pilots were overweight, with 6.4% being obese, and significant proportions presenting with high relative adiposity, abdominal obesity, hypertension, hypercholesterolemia, and hyperglycemia. Wilson et al.'s systematic review also revealed a substantial prevalence (>50%) of overweight and obesity, insufficient physical activity, elevated fatigue, and regular alcohol intake among pilots [6]. We have recently reported a higher prevalence of previous infections with herpes simplex virus 1 and *Helicobacter pylori* in APs compared to office workers, which may be associated with the prevalence of specific non-communicable diseases, including CVD, in this professional group [7]. Additionally, we have previously shown that poor sleep quality in APs correlates with lower plasma concentrations of adiponectin and elevated levels of two metabokines (fibroblast growth factor-21 and growth differentiation factor-15), potentially linking sleep disruption to a high-risk status for CVD [8].

Intima-media thickness (IMT) of the common carotid artery (CCA) is increasingly used as an indicator of generalized atherosclerosis and a surrogate marker for cardiovascular morbidity [9-11]. Notably, both body mass index (BMI) [12,13] and poor sleep quality [14,15] are associated with carotid IMT in the general population. Given the high cardiovascular risk characterizing APs, we sought to investigate, for the first time in this professional category, carotid IMT in relation to two significant risk factors for atherosclerosis development: overweight and poor sleep quality.

## Materials and methods

Study participants

The study population for this cross-sectional study consisted of 140 asymptomatic commercial APs without a history of CVD who volunteered to participate during their occupational health visits. Participants were classified as overweight if their BMI exceeded 25 kg/m^2^. Additionally, pilots with a Pittsburgh Sleep Quality Index (PSQI) score higher than five were categorized as poor sleepers [8]. The APs were then divided into four distinct groups based on their BMI and sleep quality: overweight poor sleepers (OW-PS), overweight good sleepers (OW-GS), normal weight poor sleepers (NW-PS), and normal weight good sleepers (NW-GS). Due to the limited number of female pilots, the study was restricted to male subjects [7,8]. All pilots involved in the current investigation were in apparent good physical health and held valid fitness-to-fly licenses. The study protocol was approved by the local Ethics Committee (Studio Minoretti reference number: 2022/AP/IMT). All participants were thoroughly informed about the study's objectives and provided written consent for their participation. They also agreed that the study's results might be published for scientific purposes, ensuring their anonymity was maintained.

Variables

Blood pressure and heart rate were measured using an automated digital sphygmomanometer (Lotus Global Co., Ltd., London, UK). Total serum cholesterol, low-density lipoprotein (LDL) cholesterol, high-density lipoprotein (HDL) cholesterol, and triglyceride levels were quantified in all participants using a clinical chemistry analyzer (Hitachi, Ltd., Tokyo, Japan).

IMT measurements

All examinations were conducted using an ultrasound scanner (model 128; Acuson, Mountain View, USA) equipped with a 7 MHz linear transducer with a 38 mm aperture. An ECG signal (lead II) was simultaneously recorded to synchronize image capture with the peak of the R-wave, thereby minimizing variability during the cardiac cycle. Both the right and left carotid arteries were scanned, starting from the distal part of the CCA and extending approximately 10 mm into the internal and external carotid arteries. IMT measurements were taken from the far wall of the CCA and the carotid artery bulb.

IMT definitions

IMT was defined as the distance from the leading edge of the lumen-intima interface to the leading edge of the media-adventitia interface of the far wall [16]. The average thickness of the intima-media complex was measured over 10 mm long sections of both the CCA and the carotid artery bulb. Following an established methodology [16], the primary outcome measure, referred to as the composite IMT (IMTcom), was calculated as the mean IMT of the common carotid artery and the carotid bulb from both the right and left arteries.

Data analysis

One-way analysis of variance (ANOVA) was employed to compare continuous variables among the four study groups, followed by post hoc pairwise comparisons using Bonferroni's method. Linear regression models were applied to identify the independent predictors of IMTcom in the OW-PS and OW-GS groups. Analyses were conducted using SPSS (IBM SPSS Statistics for Windows, IBM Corp., Version 20, Armonk, USA). For all tests performed, a two-sided p-value less than 0.05 was considered statistically significant.

## Results

General characteristics of the study participants

The general characteristics of the study participants are reported in Table 1. The study population consisted of 140 male airline pilots, who were categorized into four groups based on their BMI and sleep quality. The distribution of pilots among the groups was as follows: 25 (17.9%) in the OW-PS group, 36 (25.7%) in the OW-GS group, 21 (15.0%) in the NW-PS group, and 58 (41.4%) in the NW-GS group. There were significant differences among the four groups in terms of age (p<0.001), heart rate (p=0.04), total cholesterol (p=0.02), and LDL cholesterol (p=0.04). Conversely, no significant differences were observed among the four groups in terms of systolic blood pressure, diastolic blood pressure (p=0.34), HDL cholesterol (p=0.34), and triglycerides (p=0.65). Post hoc pairwise comparisons using Bonferroni's method indicated that the OW-PS group had significantly higher age, heart rate, total cholesterol, and LDL cholesterol compared to the majority of the other study groups.

**Table 1:** General characteristics of the four study groups OW-PS: overweight poor sleepers; OW-GS: overweight good sleepers; NW-PS: normal weight poor sleepers; NW-GS: normal weight good sleepers; HDL: high-density lipoprotein; LDL: low-density lipoprotein

Study group	OW-PS	OW-GS	NW-PS	NW-GS	P value
Number of pilots	25	36	21	58	-
Age, years	47 ± 5	45 ± 4	43 ± 5	44 ± 6	<0.001
Systolic blood pressure, mm Hg	130 ± 7	131 ± 8	128 ± 7	129 ± 8	0.14
Diastolic blood pressure, mm Hg	76 ± 9	75 ± 10	75 ± 9	75 ± 11	0.34
Heart rate, bpm	64 ± 8	63 ± 10	62 ± 8	60 ± 9	0.04
Total cholesterol, mmol/L	6.1 ± 0.8	5.9 ± 0.9	5.6 ± 1.0	5.7 ± 0.8	0.02
HDL cholesterol, mmol/L	1.2 ± 0.3	1.1 ± 0.5	1.2 ± 0.4	1.3 ± 0.2	0.34
LDL cholesterol, mmol/L	4.4 ± 0.6	4.2 ± 0.7	3.9 ± 0.08	3.9 ± 0.06	0.04
Triglycerides, mmol/L	1.3 ± 0.4	1.4 ± 0.8	1.2 ± 0.6	1.4 ± 0.5	0.65

IMT

Table 2 shows the results of IMT measurements in the four study groups. Significant differences were found among the four groups in terms of IMT of the common carotid artery (p=0.01) and the IMTcom (p<0.001). The mean IMTcom values for the OW-PS, OW-GS, NW-PS, and NW-GS groups were 0.95 ± 0.21 mm, 0.95 ± 0.29 mm, 0.75 ± 0.17 mm, and 0.80 ± 0.15 mm, respectively. In post hoc pairwise comparisons, the OW-PS and OW-GS groups had significantly higher IMTcom values compared to the NW-PS and NW-GS groups (p<0.001), indicating that overweight pilots, regardless of their sleep quality, had increased IMTcom. No significant differences were observed among the four groups in terms of IMT of the carotid bulb (p=0.08).

**Table 2:** Intima-media thickness data in the four study groups OW-PS: overweight poor sleepers; OW-GS: overweight good sleepers; NW-PS: normal weight poor sleepers; NW-GS: normal weight good sleepers; IMT: intima-media thickness; IMTcom: composite intima-media thickness

Study group	OW-PS	OW-GS	NW-PS	NW-GS	P value
Number of pilots	25	36	21	58	-
IMT of the common carotid artery, mm	0.9 ± 0.1	0.8 ± 0.1	0.7 ± 0.1	0.7 ± 0.1	0.01
IMT of the carotid bulb, mm	1.0 ± 0.2	1.0 ± 0.3	0.8 ± 0.1	0.9 ± 0.1	0.08
IMTcom, mm	0.95 ± 0.21	0.95 ± 0.29	0.75 ± 0.17	0.80 ± 0.15	<0.001

Independent predictors of IMTcom in OW-PS and OW-GS groups

Given the higher IMTcom values observed in both OW-PS and OW-GS groups compared to the other two study groups, linear regression models were employed to identify the independent predictors of IMTcom within these groups. In the OW-PS group, IMTcom was found to be independently associated with age (regression coefficient=0.22, p=0.01) and LDL cholesterol (regression coefficient=0.15, p=0.03). Similar associations were observed in the OW-GS group, where age (regression coefficient=0.19, p=0.02) and LDL cholesterol (regression coefficient=0.16, p=0.03) were also independently linked to IMTcom.

## Discussion

This cross-sectional study in a sample of 140 APs yielded four principal findings. First, 61 pilots had a BMI>25 kg/m^2^ and 46 had a PSQI score higher than five, yielding a prevalence of overweight and poor sleep quality of 43.6% and 32.9%, respectively. Second, after stratification of the sample into four groups based on their BMI and sleep quality, we found that the OW-PS group showed the highest burden of cardiovascular risk factors as reflected by a higher heart rate, total cholesterol, and LDL cholesterol compared to most of the other study groups. Third, we found that overweight pilots, regardless of their sleep quality, had increased IMTcom. Fourth, we observed that age and LDL cholesterol were independent predictors of IMTcom values in both the OW-PS and OW-GS groups.

The prevalence of both overweight and poor sleep quality in our sample of commercial pilots is largely consistent with previous literature. A study involving 1198 Brazilian commercial APs found that 53.7% were overweight [17], and a systematic review reported a substantial occurrence of over 50% for overweight and obesity in this occupational group [6]. Poor sleep quality is also highly prevalent among APs. Using objective assessments, Abdelaziz et al. [18] reported that 66.7% of pilots had irregular sleep patterns and 41.7% had poor sleep efficiency. Another study involving Gulf Cooperation Council commercial APs reported that 34.1% experienced excessive daytime sleepiness and 45.1% had fallen asleep at the controls at least once [19]. Given the known adverse role of both overweight [12,13] and poor sleep [14,15] on cardiovascular health, it is unsurprising that the OW-PS group in our study had an increased prevalence of well-established adverse cardiovascular risk factors, including higher heart rate, total cholesterol, and LDL cholesterol. However, this study was the first to refine the cardiovascular risk assessment in APs by measuring carotid IMT, a non-invasive, reproducible measurement obtained by ultrasound to assess subclinical atherosclerosis, which has been shown to reliably predict future adverse cardiovascular disease events at the population level [9,10]. The increased IMTcom observed in overweight pilots, irrespective of their sleep quality, suggests that BMI is a critical factor in the development of atherosclerosis in this population. Furthermore, the independent association of age and LDL cholesterol with IMTcom in both the OW-PS and OW-GS groups emphasizes the significance of these factors in the pathogenesis of subclinical atheromatous vascular disease. These findings suggest that interventions focused on reducing LDL cholesterol levels and managing age-related cardiovascular risk factors could be advantageous in mitigating the risk of vascular complications in overweight pilots. Notably, our results are consistent with the findings reported by Radjen et al. [20] in a study involving 179 military pilots who underwent measurements of the CCA IMT during their routine annual medical examinations. Their logistic regression analyses revealed that total cholesterol and BMI were independent predictors of CCA IMT, while HDL cholesterol, triglycerides, pulse pressure, and smoking were not [20]. Taken together, these findings, in conjunction with our own, underscore the critical importance of controlling dyslipidemia and overweight status to improve cardiovascular health in aviation personnel.

Our study has several notable strengths. To our knowledge, this is the first investigation to provide ultrasound-based measures of subclinical atherosclerosis in commercial APs, offering valuable insights into this understudied area in aviation medicine. Furthermore, assessing IMT at both the CCA and the carotid artery bulb enhances the robustness of our findings. Despite the valuable insights gained from this study, several limitations must be acknowledged. A fundamental caveat of cross-sectional studies is their inability to establish causation, but only associations between exposure and outcomes. The relatively small sample size further underscores the need for validation of our findings in larger cohorts. Moreover, the exclusion of female participants restricts the generalizability of our conclusions to women in the aviation industry. To elucidate the clinical implications of our results, future longitudinal studies are essential. This future research endeavor should aim to determine whether the increased IMT observed in the OW-PS and OW-GS groups may serve as a predictor for the risk of major adverse cardiovascular events in this population.

## Conclusions

This study highlights the significant prevalence of overweight and poor sleep quality among commercial pilots, which is significantly associated with increased IMT, a measure of subclinical atherosclerosis. Interventions focused on reducing LDL cholesterol levels and managing age-related cardiovascular risk factors could be advantageous in mitigating the risk of atherosclerotic vascular disease in overweight pilots. Future longitudinal studies are essential to determine the predictive value of increased IMT for major adverse cardiovascular events in this population and to inform evidence-based strategies for improving cardiovascular health in aviation personnel.
